# Molecular epidemiology and drug resistance patterns of *Mycobacterium tuberculosis* complex isolates from university students and the local community in Eastern Ethiopia

**DOI:** 10.1371/journal.pone.0198054

**Published:** 2018-09-17

**Authors:** Abiyu Mekonnen, Matthias Merker, Jeffrey M. Collins, Desalegn Addise, Abraham Aseffa, Beyene Petros, Gobena Ameni, Stefan Niemann

**Affiliations:** 1 Department of Microbial, Cellular and Molecular Biology, Addis Ababa University, Addis Ababa, Ethiopia; 2 Molecular and Experimental Mycobacteriology, Research Center Borstel, Borstel, Germany; 3 Division of Infectious Diseases, Department of Medicine, Emory University School of Medicine, Atlanta, Georgia, United States of America; 4 Ethiopian National Tuberculosis Reference laboratory, Ethiopian Public Health Institute, Addis Ababa, Ethiopia; 5 Armauer Hansen Research Institute, Addis Ababa, Ethiopia; 6 Aklilu Lemma Institute of Pathobiology, Addis Ababa University, Addis Ababa, Ethiopia; 7 German Center for Infection Research, Partner Site Borstel, Borstel, Germany; St Petersburg Pasteur Institute, RUSSIAN FEDERATION

## Abstract

**Background:**

Previous studies suggest the burden of pulmonary tuberculosis (PTB) in Ethiopia may be greater in university students relative to the overall population. However, little is known about the transmission dynamics of PTB among students and members of the communities surrounding university campuses in Eastern Ethiopia.

**Methods:**

A cross sectional study was conducted in Eastern Ethiopia among prevalent culture-confirmed PTB cases from university students (n = 36) and community members diagnosed at one of four hospitals (n = 152) serving the surrounding area. Drug susceptibility testing (DST) was performed on *Mycobacterium tuberculosis* complex (MTBC) isolates using BD Bactec MGIT 960 and molecular genotyping was performed using spoligotyping and 24-loci MIRU-VNTR. MTBC strains with Identical genotyping patterns were assigned to molecular clusters as surrogate marker for recent transmission and further contact tracing was initiated among clustered patients.

**Results:**

Among all study participants, four MTBC lineages and 11 sub-lineages were identified, with Ethiopia_3 (Euro-American lineage) being most common sub-lineage (29.4%) in both cohorts and associated with strain clustering (*P* = 0.016). We further identified 13 (8.1%) strains phylogenetically closely related to Ethiopia_3 but with a distinct Spoligotyping pattern and designated as Ethiopia_4. The clustering rate of MTBC strains was 52.9% for university students and 66.7% for community members with a Recent Transmission Index (RTI) of 17.6% and 48.4%, respectively. Female gender, urban residence, and new TB cases were significantly associated with strain clustering (*P*<0.05). Forty-eight (30%) of the study participants were resistant to one or more first line anti TB drugs, three patients were classified as multidrug resistant (MDR).

**Conclusion:**

We found evidence for recent transmission of PTB among Ethiopian university students and the local community in Eastern Ethiopia, mainly linked to strains classified as Ethiopia_3 sub lineage. Drug resistance didn’t have a major impact on recent transmission but comprehensive molecular surveillance in combination with drug resistance profiling of MTBC strains is desirable to better characterize TB transmission dynamics in high risk congregate living environments such as university campuses and guide regional TB control programs.

## Background

Tuberculosis (TB) remains a major threat to public health worldwide [1], with an estimated 10.4 million cases in 2016 [2]. Ethiopia is one of the fourteen countries with the highest TB burden with an estimated annual incidence rate of 177/100,000 population [2].

Designing public health programs to decrease TB transmission among high step to lower TB incidence rates is an essential component of the public health program in Ethiopia. However, current epidemiologic tools such as contact investigations provide incomplete information about the primary drivers of TB transmission [3].

Routine molecular characterization of *Mycobacterium tuberculosis* (MTBC) strains among incident pulmonary TB (PTB) cases offer public health officials the ability to more easily identify TB outbreaks and characterize ongoing transmission [4]. Molecular strain typing (genotyping), using the 24-loci Mycobacterial Interspersed Repetitive Units-Variable Number Tandem Repeat (MIRU-VNTR) technique in combination with Spacer Oligo Nucleotide typing (Spoligotyping) is widely used for this purpose [5, 6, 7, 8], and has been shown to more effectively inform public health interventions [9]. In addition, certain MTBC lineages (e.g. sub lineage 2 [Beijing]) have been associated with increased pathogenicity and resistance to specific drugs and tracking the transmission of these lineages may also provide information about the risk for drug resistance in regions where they are prevalent [8].

Recent studies of university students in central and Eastern Ethiopia suggest TB incidence may be higher on school campuses relative to country-wide TB incidence rates [10, 11]. Rapid increases in university enrollment in Ethiopia has led to crowded congregate living environments on college campuses with the potential to facilitate TB transmission. However, the most prevalent circulating MTBC strains and the relative contribution of recent TB transmission among pulmonary TB cases, both on campus and in surrounding communities, are unknown. We studied university students and surrounding community members diagnosed with PTB at three universities and four hospitals in Eastern Ethiopia to determine the genotypic characteristics, transmission dynamics and drug resistance patterns of MTBC strains circulating in the region.

## Materials and methods

### Study design

A cross-sectional study was conducted among students diagnosed with PTB while attending one of three Eastern Ethiopian universities and community members diagnosed with PTB at one of four hospitals located near these university campuses. The study area included three regional states and one administration: Oromia, Somali and Harari regional states and Dire Dawa City administration. According to the Central Statistics agency of Ethiopian population projection values of 2017, the population of Harari and Somali regional states were 246,000 and 5, 748, 998, respectively; whereas that of Dire Dawa city administration and Haramaya district were 466,000 and 361,787, respectively [12]. All PTB cases included in the study were bacteriologically confirmed by a positive sputum culture for MTBC.

Students with PTB were identified through active case finding between May 2016 and April 2017. All full-time students attending Haramaya University, Dire Dawa University and Jigjiga University were screened for symptoms of PTB through dormitory-to-dormitory visits using WHO TB screening document [13]. For students with a positive symptom screen, two spot sputum samples and relevant clinical and socio demographic data were collected. One sputum sample was processed for Acid fast bacilli (AFB) smear microscopy and the other one was transported to Harari Health Research and Regional Laboratory for MTB culture. Because sputum cultures are not routinely obtained in students diagnosed with PTB at student health centers, only previously undiagnosed, prevalent PTB cases were enrolled in this study.

Community TB cases were enrolled from hospitals serving the geographic areas surrounding the universities from January to April 2017. Participants were enrolled from Haramaya district hospital (Haramaya), Hiwot Fana specialized university hospital (Harar), Dil Chora hospital (Dire Dawa) and Karamara hospital (Jigjiga). Persons presenting to these facilities with symptoms of PTB and found to have a positive AFB sputum smear were approached to participate in the study. Persons giving consent for study participation were administered a standard questionnaire to collect information about relevant clinical and sociodemographic data. An early morning sputum sample was collected from each smear positive patient and stored at -20°c until transported to Harari Health Research and Regional Laboratory for MTB culture.

### Laboratory methods

All sputum specimens were cultivated on LJ (BBL^™^ Lowenstein-Jensen) media at Harari Health Research and Regional laboratory following standard operating procedures. Isolates were then transported to the National TB reference laboratory in Addis Ababa, where they were reactivated and phenotypic Drug Susceptibility Testing (DST) was performed using the MGIT SIRE kit at a critical concentration of streptomycin (STM) 1 μg, Isoniazid (INH) 0.1 μg, Rifampicin (RIF) 1 μg and Ethambutol (EMB) 5 μg on liquid Mycobacterium Growth Indicator Tube system (MGIT) 960 as previously described [14].

DNA from MTBC isolates was extracted and transported to Borstel Molecular Mycobacteriology laboratory, Germany for genotype analysis. Molecular characterization of all isolates was conducted using Spoligotyping [15] and 24- loci MIRU-VNTR customized kits (Genoscreen, Lilli, France) [16]. Participants for whom a valid genotype could not be obtained were excluded. This included evidence of a mixed infection or laboratory cross-contamination as indicated by double alleles at two or more loci during MIRU/VNTR typing and two or more loci with missing data following at least two independent PCR amplifications. Reasons for missing loci included insufficient DNA concentration [17] and nucleotide polymorphisms in the sequence complementary to the PCR primers [18]. Samples with no PCR amplicon at only one locus in the 24-loci MIRU/VNTR analysis were included for further analysis by considering missing data at the respective locus [19].

### Data analysis

**Fig 1:**
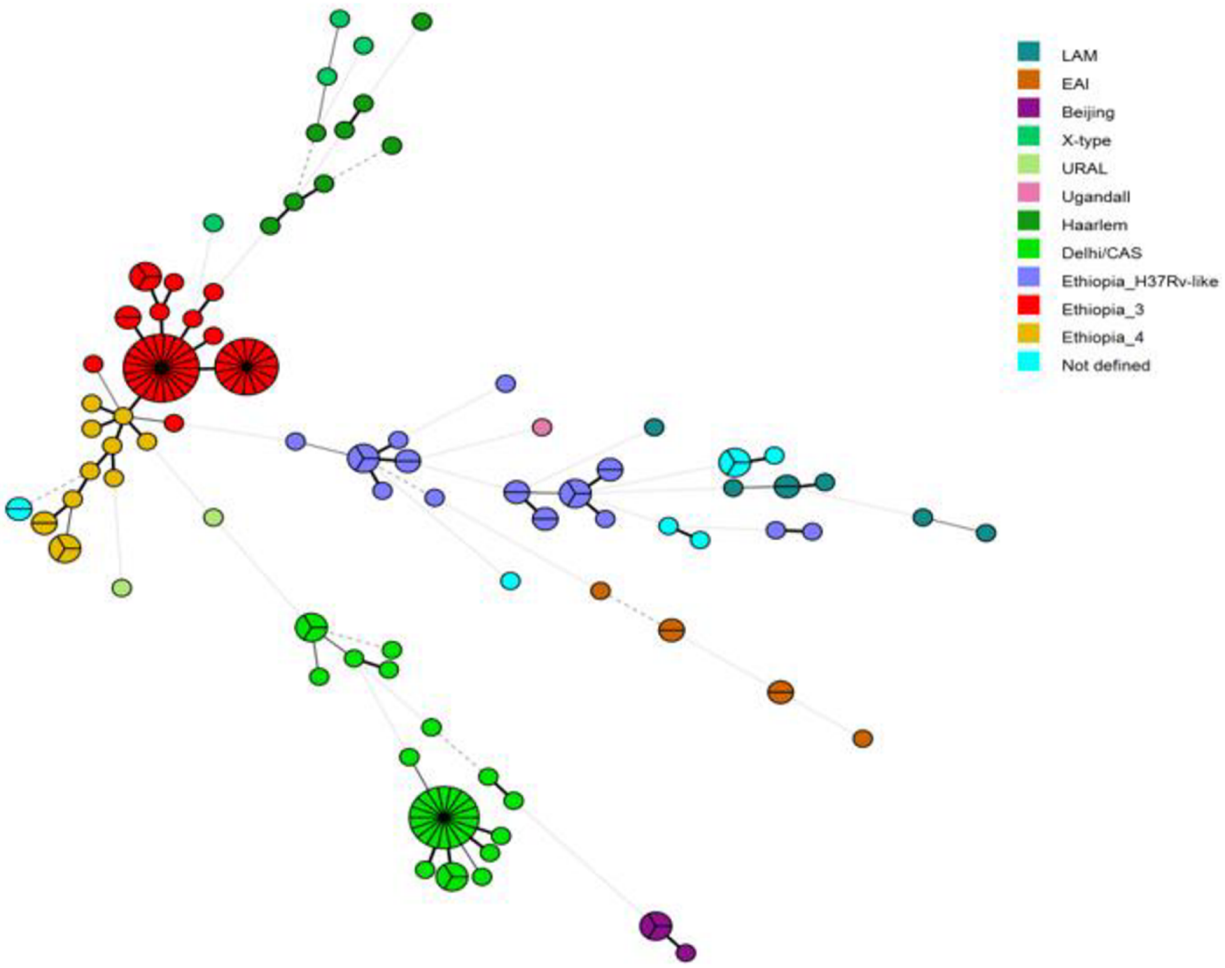
Minimum spanning tree based on 24-loci MIRU-VNTR profiles of 160 MTBC isolates from Eastern Ethiopia. The identified sub-lineages are color-coded. Branch lengths are proportional to number of MIRU-VNTR loci differences between two nodes (<1 locus, solid thick lines; 2–3 loci, solid thin lines; >4 loci, dashed line). Node size is proportional to the number of isolates with identical MIRU-VNTR profiles. EAI: East Africa India, LAM = Latin American Mediterranean.

MTBC genotypes were classified in a phylogenetic tree (based on 24-loci MIRU-VNTR profiles) in relation to a MTBC reference collection hosted on the MIRU-VNTRplus website (available at www.miru-vntrplus.org) and considering genotype specific Spoligotyping patterns [20]. Minimum Spanning Trees (MST) were calculated with BioNumerics (Version 7.5; Applied Maths, Sint-Martens-Latem, Belgium) as recommended by the manufacturer (available at http://applied-maths.com). A dendrogram was generated using the Unweighted Pair Group Method with Arithmetic averages (UPGMA) based on the 24-loci MIRU-VNTR profiles. The UPGMA tree was further processed using EvolView, an online visualization and management tool for customized and annotated phylogenetic trees [21] (Fig 1).

Samples with complete spoligotyping and MIRU-VNTR-24 results were used for clustering analysis. Spoligotypes common to more than one strains were designated as shared types (ST) and were assigned a shared international type number (SIT) according to the international spoligotype database SpolDB4 [22]. Basic strain classification and MLVA MTBC 15–9 nomenclature assignment was done using the MIRUVNTRplus database [20]. A cluster was defined as two or more MTBC isolates sharing identical 24-loci MIRU/VNTR and spoligotyping patterns. The Recent Transmission Index (RTI) was calculated as number of clustered patients minus number of clusters divided by total number of patients as described previously [17].

Data were entered and analyzed using IBM SPSS version 23 statistical package software. Logistic regression was used to estimate the strength of association between strain clustering and different variables. A Chi-square test was used for bivariate analysis of categorical variables. P-values <0.05 were considered as statistically significant. Those factors significantly associated with clustering in the univariate analysis were included in the multivariate regression model.

### Ethical considerations

The study was ethically reviewed and approved by Addis Ababa University, College of Natural Sciences Research Ethics Review Board. Written informed consent that included information about the risks and benefits of the study was a prerequisite for study participation.

## Results

### Study population

A total of 35,344 students (92.2% of students enrolled in the three universities) were screened for symptoms of PTB. Of students screened, 1,097 had a positive symptom screen and 36 were culture positive for MTBC. A total of 1,523 PTB cases were diagnosed at that four hospitals in the community, of which 183 had a positive AFB sputum smear, and 171 agreed to participate in the study and submitted sputum samples; finally, 152 had a positive sputum culture for MTBC. Molecular genotyping was performed on all 188 MTBC isolates using Spoligotyping and 24-loci MIRU-VNTR techniques. Of these, 28 isolates were excluded for technical reasons; two isolates were found to be a mixed infection as indicated by double alleles at two or more loci during MIRU-VNTR typing and 26 isolates had two or more loci missing following PCR amplification. The final genotypic analysis included 160 MTBC strains: 34 from university students and 126 from the local community. The majority of the study participants were male (80%) and rural residents (60.6%). Forty-six (28.7%) were previously treated for TB, and 10.6% were HIV-positive (Table 1).

**Table 1:** Characteristics of university students and members of the surrounding community diagnosed with pulmonary tuberculosis, Eastern Ethiopia.

		Students^a^ n = 34	Local Community^b^ n = 126	Total n = 160	P-value
Gender n (%)	M	33 (97.1)	95 (75.4)	128 (80.0)	0.005
Age group n (%)	<18	0 (0)	16 (12.7)	16 (10.0)	NA
	18–24	33 (97.1)	27 (21.4)	60 (37.5)	
	25–34	1 (2.9)	40 (31.7)	41 (25.6)	
	35–44	0 (0)	23 (18.3)	23 (14.4)	
	≥ 45	0(0)	20 (15.9)	20 (12.5)	
Location/Region n (%)	Haramaya/Harar	18 (53.0)	46 (36.5)	64 (40.0)	0.177
	Dire Dawa	6 (17.6)	38 (30.2)	44 (27.5)	
	Jigjiga	10 (29.4)	42 (33.3)	52 (32.5)	
Residence n (%)	Urban	3 (8.8)	60 (47.6)	63 (39.4)	NA
	Rural	31 (91.2)	66 (52.4)	97 (60.6)	
Previous Rx for TB n (%)	Yes	6 (17.6)	40 (31.7)	46 (28.7)	0.107
HIV status n (%)	Positive	2 (5.9)	15 (11.9)	17 (10.6)	0.614
Clustering n (%)	Yes	18 (52.9)	84 (66.7)	102 (63.8)	0.140

^a^. Prevalent culture-positive TB cases diagnosed through active case finding at Haramaya University, Dire Dawa University or Jigjiga University

^b^. Acid-fast bacilli sputum smear positive pulmonary TB cases diagnosed at Haramaya district hospital (Haramaya), Hiwot Fana specialized university hospital (Harar), Dil Chora hospital (Dire Dawa) or Karamara hospital (Jigjiga)

### Phylogenetic analysis of *M*. *tuberculosis* strains

The two predominant MTBC lineages in this study were lineage 4 (Euro-American, 71.3%) and lineage 3 (Delhi-CAS, 22.5%). MTBC strains classified as lineage 1 (East African Indian), and lineage 2 (Beijing) were only identified in six and four patients, respectively (Table 2).

**Table 2:** Phylogenetic sub-lineages and drug susceptibility patterns of *Mycobacterium tuberculosis* isolates from university students and members of the surrounding community diagnosed with pulmonary tuberculosis in Eastern Ethiopia.

		Students^a^ n = 34	Local Community^b^ n = 126	P-Value
Phylogenetic Sub lineage n (%)	Ethiopia_3	10 (29.4)	38 (30.2)	0.074
	Ethiopia_4	1 (2.9)	12 (9.5)	
	Ethiopia_H37 Rv like	7 (20.6)	15 (11.9)	
	Delhi/CAS	7 (20.6)	29 (23.0)	
	Beijing	0 (0)	4 (3.2)	
	LAM	1 (2.9)	6 (4.8)	
	Haarlem	1 (2.9)	7 (5.6)	
	X-type	0 (0)	4 (3.2)	
	EAI	0 (0)	6 (4.8)	
	UgandaII	1 (2.9)	0 (0)	
	URAL	1 (2.9)	1 (0.8)	
	Not defined	5 (14.7)	4 (3.2)	
Streptomycin n (%)	Resistant	7 (20.6)	17 (13.5)	0.304
Isoniazid n (%)	Resistant	6 (17.6)	15 (11.9)	0.379
Rifampin n (%)	Resistant	0 (0)	9 (7.1)	0.109
Ethambutol n (%)	Resistant	0 (0)	9(7.1)	0.109
Resistance to ≥1 Anti-TB drugs n (%)	Yes	11 (32.4)	37 (29.4)	0.734
MDR n (%)	Yes	0 (0)	3 (2.4)	0.364

MDR: Multidrug resistant, EAI: East-African Indian; LAM: Latin American Mediterranean;

^a^. Prevalent culture-positive TB cases diagnosed through active case finding at Haramaya University, Dire Dawa University or Jigjiga University

^b^. Acid-fast bacilli sputum smear positive pulmonary TB cases diagnosed at Haramaya district hospital (Haramaya), Hiwot Fana specialized university hospital (Harar), Dil Chora hospital (Dire Dawa) or Karamara hospital (Jigjiga)

The most prevalent MTBC genotypes within the Euro-American super-lineage were Ethiopia_3 (29.4%) and Ethiopia-H37Rv-like (12.8%), both previously described among Ethiopian patients with PTB [17], and TB lymphadenitis [23]. Based on the phylogenetic structure/topology (UPGMA- and MST-based), we further termed the third largest monophyletic lineage 4 group “Ethiopia_4” (8.1%) accordingly (Fig 1). Ethiopia_4 strains are closely related to Ethiopia_3 strains but with a distinct Spoligotyping pattern. Both, Ethiopia_3 and Ethiopia_4 strains, have a shared common ancestor with TUR-genotype strains, but with unique Spoligotyping patterns, justifying their own nomenclature in the context of the molecular epidemiology in Ethiopia.

MTBC strains classified as Beijing, East Africa India (EAI), or X-type were isolated exclusively from community members and not identified among university students. Beijing strains were only isolated from members of the surrounding Dire Dawa community. Nine MTBC isolates (5.6%) could not be classified within any of the known genotypes but were part of lineage 4 with an unknown sub-group and are labeled “Not defined”.

### Molecular MTBC clusters and associated risk factors

**Fig 2:**
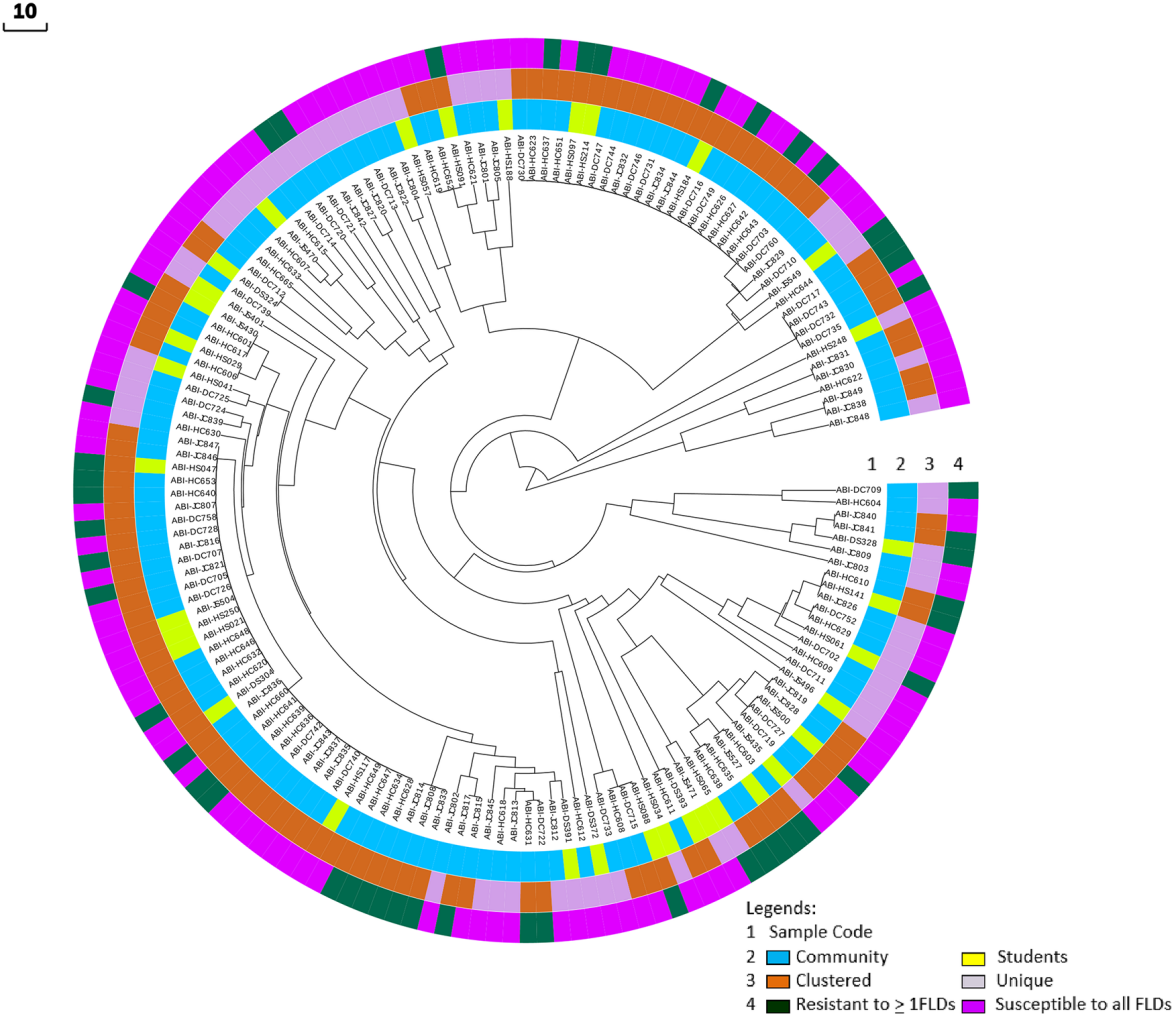
Visualization of PTB cases using a radial UPGMA tree based on 24-loci MIRU-VNTR data. Ring 4 shows drug resistance categories per isolate: fully susceptible (pink), at least one first-line drug resistance (dark green). Ring 3 shows whether cases were part of a genetic cluster (orange) or were unique (purple). Ring 2 differentiates students (yellow) from community members (blue).

The overall cluster rate of MTBC strains derived from all patients was 63.8%, including 21 clusters with 2 to 22 patients. Twelve clusters contained at least one university student. The overall Recent Transmission Index (RTI) was 50.6% (Table 3). Female gender (P = 0.004), urban residence (P = 0.012) and newly diagnosed PTB cases (P = 0.001) were all significantly associated with being part of a cluster. Additionally, MTBC strains classified as Ethiopia_3 were significantly more likely to be part of a cluster compared to other MTBC genotypes/sub lineages (Table 4, Fig 2).

**Table 3:** Clustering rate and Recent Transmission Index (RTI) analysis using Spoligotyping and MIRU-VNTR 24-loci methods for local community, university students and all study participants, Eastern Ethiopia.

Methods	Source of MTBC isolate	No. of different patterns	Unique Patterns	Number of clusters	Number of isolates in cluster	Clustering Rate	RTI
Spoligotyping	Students	18	6	12	28	82.4%	26.5%
	Local community	38	12	26	114	90.5%	69.8%
	Both students and local community	40	18	22	142	88.7%	75%
24-loci MIRU-VNTR	Students	28	16	12	18	52.9%	17.6%
	Local community	63	38	25	88	69.8%	50%
	Both students and local community	77	55	22	105	65.6%	51.9%
Spoligotyping + 24-loci MIRU-VNTR	Students	28	16	12	18	52.9%	17.6%
	Local community	65	44	21	84	66.7%	48.4%
	Both students and local community	79	58	21	102	63.8%	50.6%

**Table 4:** Multivariable logistic regression analysis of factors associated with *Mycobacterium tuberculosis* strain clustering among university students and the local community in Eastern Ethiopia.

Variable		Genotype		AOR (95% C.I)	P-value
		Clustered n (%)*	Unique n (%)*		
Gender	Male	75 (58.6)	53 (41.4)	1	
	Female	27 (84.4)	5 (15.6)	7.14 (1.85, 27.52)	0.004
Residence location	Urban	48 (76.2)	15 (23.8)	4.19 (1.36, 12.89)	0.012
	Rural	54 (55.7)	43 (44.3)	1	
Previous DX for TB	No	83 (72.8)	31 (27.2)	6.50 (2.14, 19.79)	0.001
	Yes	19 (41.3)	27 (58.7)	1	
Isoniazid	Susceptible	84 (60.4)	55 (39.6)	1	
	Resistance	18 (85.7)	3 (14.3)	7.81 (0.75, 81.69)	0.086
Any resistance to FLDs	No	62 (55.4)	50 (44.6)	1	
	Yes	40 (83.3)	8 (16.6)	3.23 (0.80, 13.01)	0.100
Genotype	Ethiopia_3	41 (87.2)	7 (12.8)	13.37 (1.63,109.6)	0.016
	Ethiopia_4	7	6	1.11 (0.12, 10.65)	0.931
	Ethiopia_H37 Rv like	14	8	1.39 (0.17, 11.49)	0.760
	Delhi/CAS	25	11	3.49 (0.48, 25.66)	0.219
	Beijing	4	0	NA	NA
	LAM	2	5	0.10 (0.01, 1.48)	0.094
	Haarlem	0	8	NA	NA
	X-type	0	4	NA	NA
	EAI	4	2	3.26 (0.19, 55.52)	0.414
	UgandaII	0	1	NA	NA
	URAL	0	2	NA	NA
	Not defined	5	4	1	

*Percentage is calculated from row total; FLDs: first line anti-TB drugs

There was no a statistically significant difference in the proportion of clustered strains between university students and local community (p = 0.142) (Table 1). With regard to the university cohort, 18/34 (52.9%) patients were part of a molecular cluster. Seventeen (94%) lived in an area at least 400 km away prior to attending university. There were two clusters with multiple student cases. One cluster contained three students, all of whom were attending Haramaya University. Two of the three students shared a common area of study, but none were living in the same dormitory or building. The other cluster contained five student cases, with three of the students attending Haramaya University and the other two attending Jigjiga and Dire Dawa Universities, respectively. None of the students in this cluster shared a clear epidemiologic link. Of the eight students clustered with other student cases, six (75%) reported recent exposure to someone with a cough, but none had a known exposure to an active TB case on campus. The RTI among students was 17.6% (Table 3).

### Patterns of *M*. *tuberculosis* drug resistance

Forty-eight (30%) of the study participants were resistant to one or more first line anti TB drugs. Among the first line anti-TB drugs examined, resistance to streptomycin and isoniazid was most common at 15%, and 13.1%, respectively. Rates of resistance to at least one first line anti-TB drug were similar in students and the community (32.4% vs 29.4%, p = 0.734) (Table 2). Few participants had multidrug resistant (MDR) TB, with three cases occurring in community members (infected with Ethiopia_3, Ethiopia H37Rv-like, and LAM strains, respectively) and none occurring among university students. Ethiopia_3 strains were observed with a higher proportion (9.4%) of resistance to at least one first-line drug compared to other MTBC genotypes (Table 5).

**Table 5:** Patterns of drug resistance to first line anti-TB drugs by *Mycobacterium tuberculosis* sub-lineages among university students and the local community in Eastern Ethiopia.

	Streptomycin n (%)*	Isoniazid n (%)*	Rifampin n (%)*	Ethambutol n (%)*	Resistance to any first line drugs n (%)*	Multidrug resistance n (%)*
Ethiopia_3 (n = 48)	8 (16.7)	8 (16.7)	3 (6.3)	1 (2)	15 (31.3)	1 (2)
Ethiopia_4 (n = 13)	3 (23)	1 (7.7)	1 (7.7)	4 (30.8)	6 (46.2)	0
Ethiopia_H37 Rv like (n = 22)	2 (9)	5 (22.7)	1 (4.5)	2 (9)	8 (36.4)	1 (4.5)
Delhi/CAS (n = 36)	5 (13.9)	2 (5.6)	1 (2.8)	1 (2.8)	9 (25)	0
Beijing (n = 4)	2	1	1	0	3	0
LAM (n = 7)	1 (14.3)	2 (28.6)	1 (14.3)	1 (14.3)	3 (42.9)	1 (14.3)
X-type (n = 4)	1	1	1	0	2	0
Not defined (n = 9)	2 (22.2)	1 (11.1)	0	0	2 (22.2)	0
Total (n = 160)	24 (15.0)	21 (13.1)	9 (5.6)	9 (5.6)	48 (30.0)	3 (1.9)

*Percentage calculated from the total number of isolates in each sub-lineage (n) and for those sub-lineages with more than 5 strains; MDR: Multi Drug Resistance; n = number of resistance strains in the specific group.

## Discussion

By using molecular MTBC strain typing (24-loci MIRU-VNTR typing and Spoligotyping) we found an overall high cluster rate of 63.8% among all study participants suggesting that recent PTB transmission remains a major public health concern in Eastern Ethiopia. In particular, Ethiopia-3, the most prevalent MTBC sub-lineage among students and the community members, was associated with strain clustering and is considered as the main driver for recent transmission among our study cohorts.

Although the recent transmission index was lower among university students compared to community members, there were several clusters that contained multiple students on university campuses. However, all clusters with student cases also included at least one case from the community, consistent with a more complicated network of TB transmission between students and the surrounding community that it is not limited to congregate living facilities (i.e. crowded university dormitories). Nearly half of student cases had unique molecular strain types. Given most university students with active TB originated from areas at least 400 km away and only moved to university campuses relatively recently, these unique isolates may be due to reactivation of latent TB infection acquired in the student’s region of origin. Larger scale, prospective molecular surveillance of MTBC strains in the region will likely be required to more comprehensively characterize transmission dynamics of TB on and around university campuses to help design the most impactful interventions to interrupt transmission.

Molecular clusters defined by the applied genotyping methods are a surrogate marker for recent transmission and epidemiological linked cases [24]. The high clustering rate of 63.8% in this study indeed indicates that the majority of active TB cases in this study population were due to recent transmission and not reactivation. Studies from other parts of the world, including South Tawara, Kiribat [25], have shown an even higher clustering rate of 75.3%. Although there were no previous studies conducted in eastern Ethiopia using the standard combined application of 24-loci MIRU-VNTR and Spoligotyping methods, a study from Northwestern Ethiopia demonstrated a clustering rate of 45.1% [17], small, differences in estimates of recent transmission between individual studies might be explained by different living environments in the areas studied (e.g. rural vs. metropolitan setting) or other demographic differences (e.g. age, gender) [26]. However, these studies have consistently shown a large proportion of TB cases in Ethiopia are due to recent transmission.

Multivariable analysis demonstrated several demographic and clinical factors were associated with clustering. Females were more likely to be part of a cluster than their male counterparts, which is similar to previous studies from Ethiopia [27] and Botswana [28] and could be linked to an increased tendency of females spending more time in close contact with their relatives in crowded settings like market places. However, females were also underrepresented in our sample, particularly among university students, so this result should be interpreted with caution. Urban residents were more than four times more likely part of a cluster, compared to those living in rural areas, which could be a result of the dense living conditions in cities.

The predominant MTBC lineages in this study were lineage 4 followed by lineage-3; findings that are similar to other studies conducted in Ethiopia [17,19, 26]. We also demonstrate that local sub-linages such as the newly described “Ethiopia_4” and the closely related Ethiopia_3 (both part of lineage 4 and related to TUR genotype strains) dominate among Ethiopian TB cases but do not play a major role in the global TB epidemic. This might be another example of a specialized, locally adapted MTBC strain type as recently suggested by Stucki and colleagues for the lineage 4 strains [29]. However, it is also important to note that strains with the Ethiopia H37Rv-like genotype can be found in many other world regions [25, 30, 31], and shares a common ancestor with the H37Rv laboratory reference strains (MTBC lineage 4.7, 4.8, and 4.9) according to Coll and his colleagues [32].

Strains from the Ethiopia_3 sub-lineage were more likely to be part of a cluster, indicating active transmission in the study area; an observation that was also found in Northwestern Ethiopia [17] and another study from Eastern Ethiopia [26]. This finding highlights the need to conduct a large scale and more detailed characterization of the Ethiopia_3 sub-lineage in Ethiopia. Studies conducted in Northern, Northwestern and Southwestern Ethiopia have found the Dehli/CAS to be the predominant lineage [17, 23, 33, 34], which may be attributable to these regions bordering Sudan, where Dehli/CAS was the most prevalent MTBC genotype [35]. All active TB cases caused by the Beijing strain, which is associated with high virulence, multidrug resistance and increased mortality [36], originated from the Dire Dawa community, which also supports the need to monitor the disease in the region. This study did not reveal any lineage-7 isolates (also referred to as “Ethiopia_1” in previous studies [17, 23]), which is in agreement with previous work demonstrating the predominance of lineage-7 in the northern part of the country [37].

This study is subject to several limitations. Enrolled participants from communities surrounding university campuses included only smear positive PTB cases who have access to and visited the study hospitals, which may not represent all persons with PTB in the geographic regions. Because all enrolled students with PTB were diagnosed by active case finding, we were only able to enroll prevalent and previously undiagnosed PTB cases. Additionally, due to the cross-sectional nature of the study design conducted over a short period of time that clusters of TB cases both among students and members of the surrounding community might not be completely characterized and additional case clusters might have been missed. Larger, prospective studies of MTBC isolates Combining genotype data with more detailed geospatial mapping of pulmonary TB cases would be of interest to further elucidate more complex transmission dynamics of PTB in the geographic area.

## Conclusion

This study suggests recent TB transmission accounts for a significant proportion of PTB cases in the examined region of Eastern Ethiopia and is associated with Ethiopia_3 sub lineage. While there is evidence of TB transmission on university campuses, transmission among students is not limited to the university setting and has significant overlap with case clusters in the general community. Country-wide comprehensive molecular surveillance and DST profiling of MTBC strains are desirable to guide ongoing and future TB control programs.

## Supporting information

S1 Fig Neighbor joining (NJ) phylogenetic tree based on 24-loci MIRU-VNTR profiles of 160 MTBC isolates from Eastern Ethiopia (PDF) in relation to the MTBC reference collection hosted on miru-vntrplus.org. (PDF)
