# Supplementary figures and images for: Molecular epidemiology and drug resistance patterns of Mycobacterium tuberculosis complex isolates from university students and the local community in Eastern Ethiopia
Source: PLoS One. 2018 Sep 17;13(9):e0198054. doi: 10.1371/journal.pone.0198054 (PMC6141063; doi:10.1371/journal.pone.0198054)

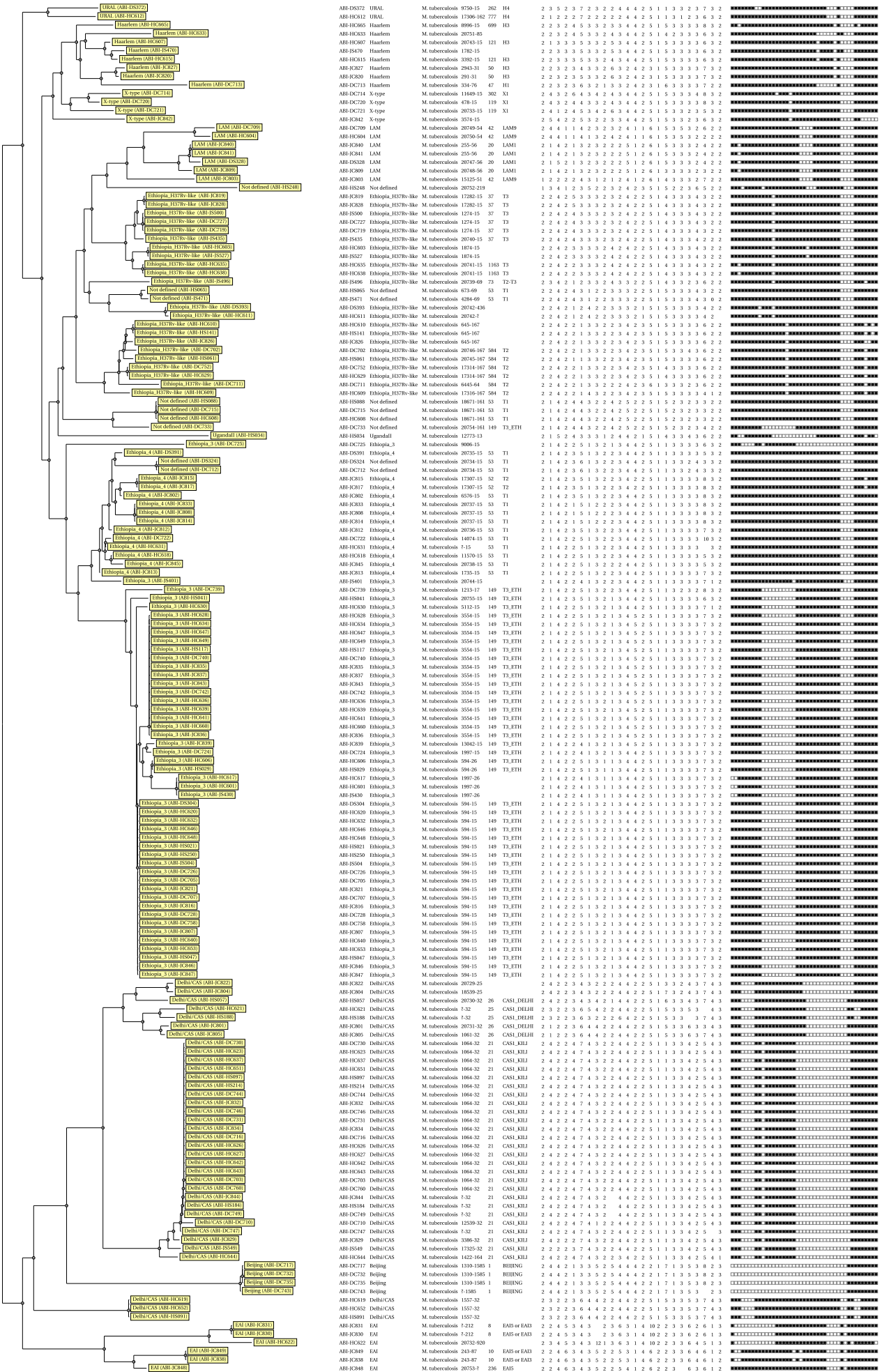

Supplement: S1 Fig — (PDF) [file pone.0198054.s001.pdf]
